# Supplementary material for: PAM-flexible Cas9-mediated base editing of a hemophilia B mutation in induced pluripotent stem cells
Source: Commun Med (Lond). 2023 Apr 19;3:56. doi: 10.1038/s43856-023-00286-w (PMC10115777; doi:10.1038/s43856-023-00286-w)
Supplement: Supplementary file 1 — Supplementary Materials [file 43856_2023_286_MOESM1_ESM.pdf]

Supplementary Materials

PAM-flexible Cas9-mediated base editing of a hemophilia B mutation in induced pluripotent stem cells

Takafumi Hiramoto, Yuji Kashiwakura, Morisada Hayakawa, Nemekhbayar Baatartsogt, Nobuhiko Kamoshita, Tomoyuki Abe, Hiroshi Inaba, Hiroshi Nishimasu, Hideki Uosaki, Yutaka Hanazono, Osamu Nureki, and Tsukasa Ohmori*

*Corresponding author: Tsukasa Ohmori, M.D., Ph.D., Department of Biochemistry, Jichi Medical University School of Medicine; 3311-1 Yakushiji, Shimotsuke, Tochigi 329-0498, Japan. Email: tohmori@jichi.ac.jp. Tel: +81-285-58-7324.

Supplementary Figures 1 – 5
Supplementary Tables 1 – 2
Supplementary Data 1 – 2 (Provided in another Microsoft Excel file)

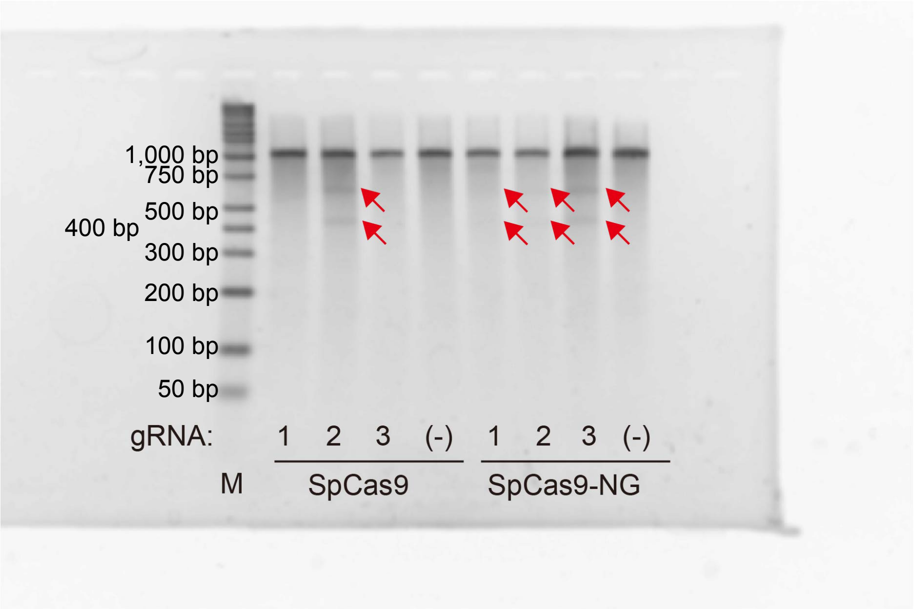

Supplementary Figure 1. Uncropped gel image of Fig. 1d.

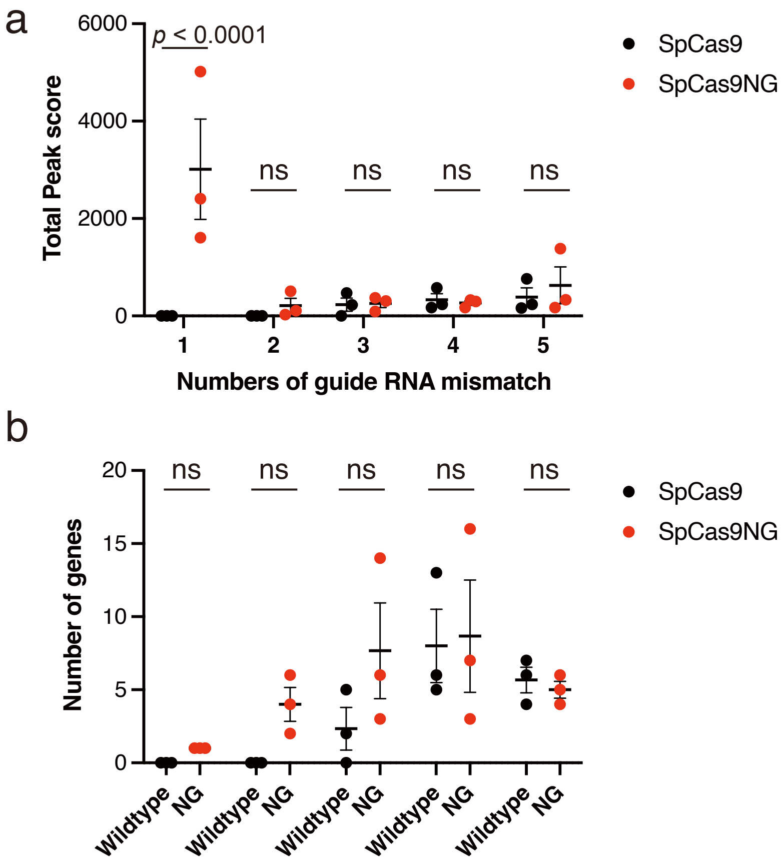

Supplementary Figure 2. GUIDE-Seq analysis to detect off-target cleavages. HEK293 cells were transduced with the plasmid expressing SpCas9 or SpCas9-NG and gRNA3 together with dsODN. GUIDE-seq was analyzed as describe in Method section. (**a**) The total peak scores at each number of mismatch from guide RNA sequence. Each dot indicates each score by wildtype SpCas9 (black) or SpCas9NG (red) with guide RNA3. (**b**) The number of genes influenced by off-target cleavages. The Data are presented as mean ± SEM or mean. Statistical significance was determined using two-tailed Student's *t* test. ns, not significant.

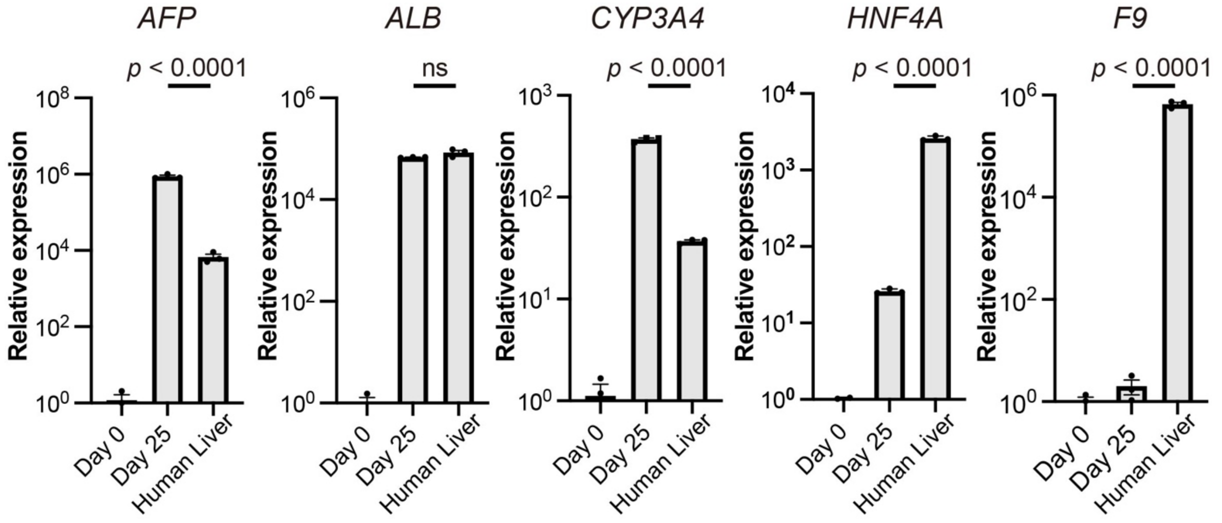

Supplementary Figure 3. Analysis of hepatocyte-specific gene expression. Relative mRNA expression of hepatocyte-specific proteins (*AFP*, *ALB*, *CYP3A4*, *HNF4A*, and *F9*) in the parental iPSCs before (day 0) and after *in vitro* hepatic lineage differentiation (day 25), and human liver RNA. Values represent mean ± SEM (n = 3). Statistical significance was determined using one-way ANOVA with post hoc Tukey's multiple comparison test. ns, not significant.

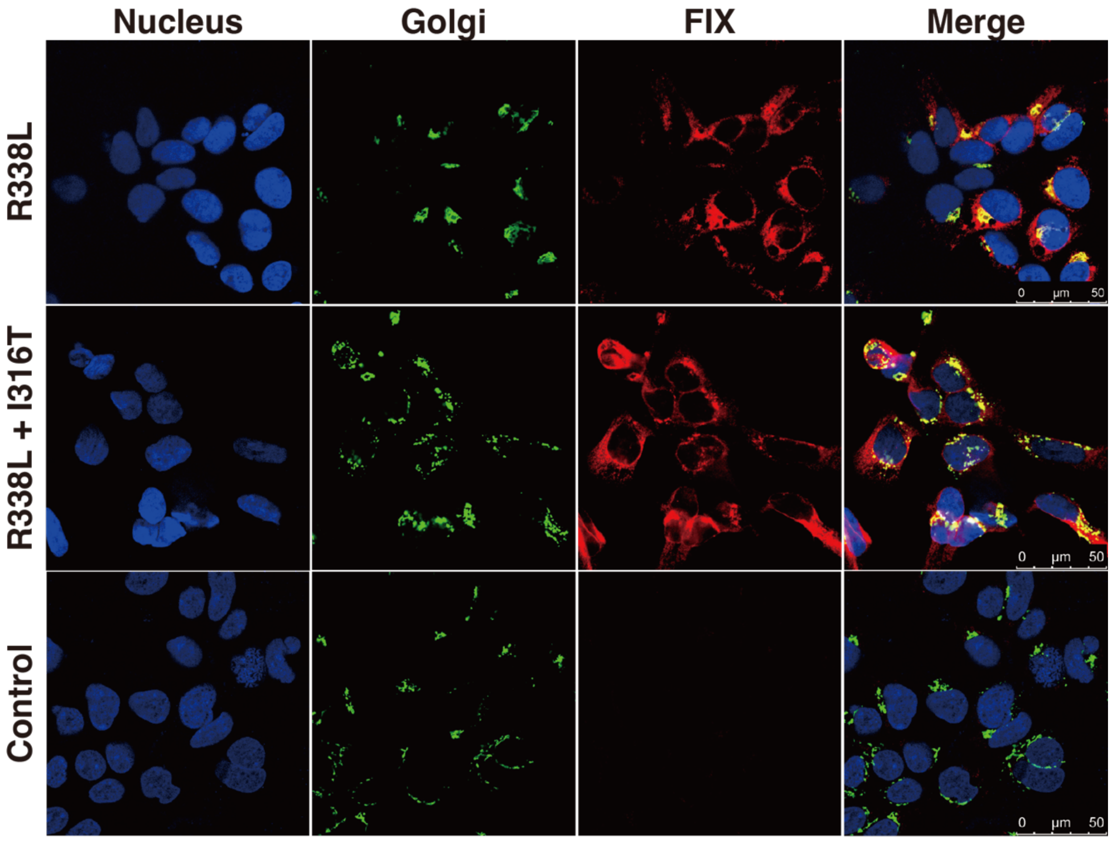

Supplementary Figure 4. The intracellular localization of FIX protein in HEK293 cells stably expressing the patient mutation. The HEK293 cells stably expressing human *F9* cDNA (R338L mutation) and the patient *F9* cDNA (R338L + I316T mutations) were established by plasmid transfection. The immunohistochemical staining the cells were observed and photographed using a confocal microscope (Leica TCS SP8). Red, FIX; green, Golgi; blue, DAPI. Control, HEK293 cells without transfection. The scales are indicated in the figures.

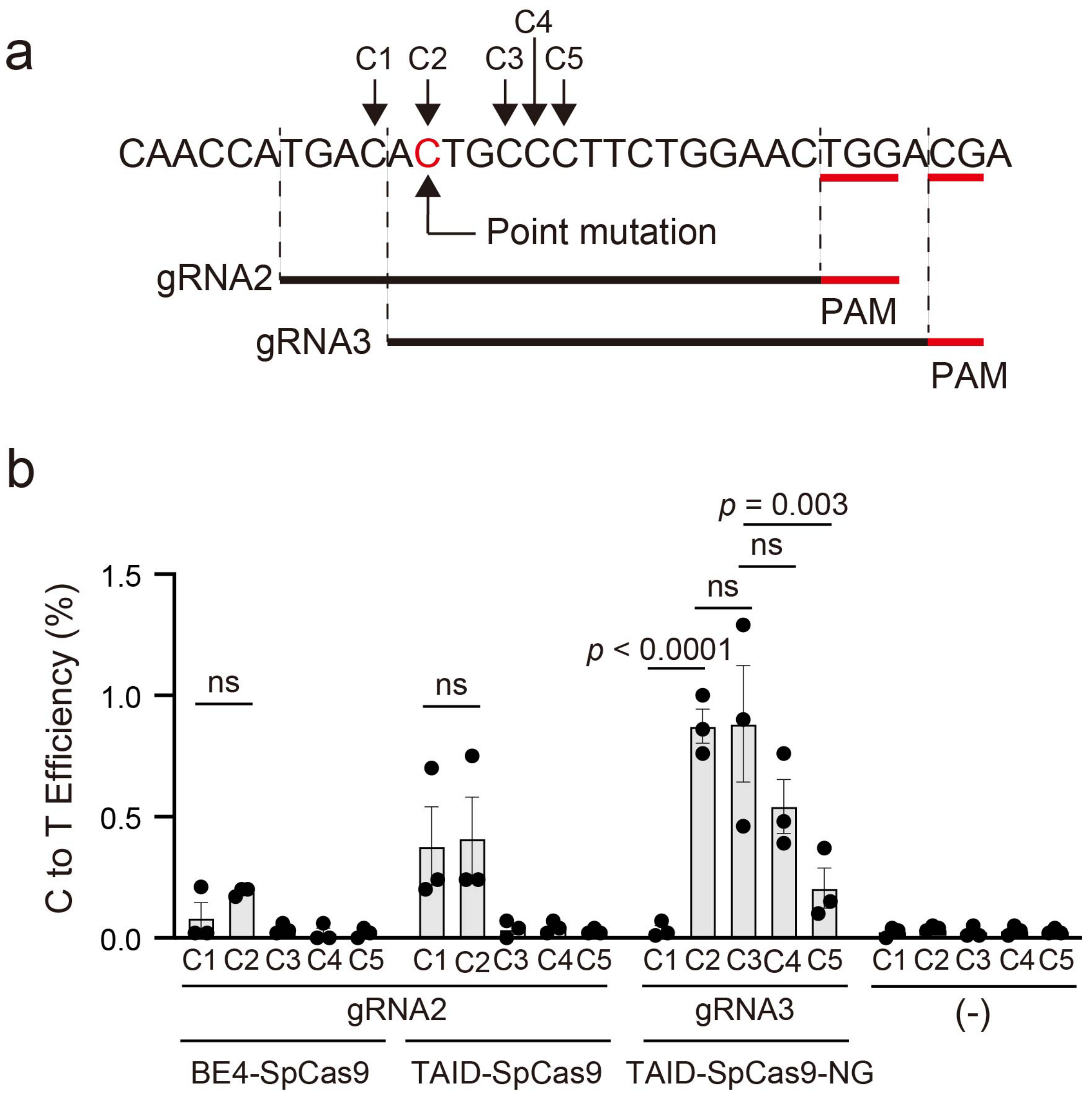

Supplementary Figure 5. Analysis of bystander activities near the target regions. The frequencies of C to T conversion near the mutation of the severe hemophilia B patient-derived iPSCs after transduction with BE4-SpCas9, TAID-SpCas9, or TAID-SpCas9-NG plasmid harboring gRNA2 or gRNA3. The frequency of C to T conversion was examined using next-generation sequencing. (**a**) Five candidate target cytidines exist 5' sequences of guide RNA2 or guide RNA3. (**b**) The frequencies of C to T conversion by the transduction of guide RNAs with BE4 or TAID based on wildtype SpCas9 or SpCas9NG. The Data are presented as mean ± SEM. Statistical significance was determined using two-way ANOVA with post hoc Tukey's multiple comparison test. ns, not significant.

Supplementary Table 1. PCR primers and dsODN sequences

Name	Sequence (5'-3')	Purpose
I316T FW	5'-AAAGGGGTGAGGATACTTGAAAT-3'	Surveyor Assay and sequencing
I316T RV	5'-TTCCTCTAGTGGTTCCATTTTCTA-3'	Surveyor Assay and sequencing
NGS-FW1	5'-TCGTCGGCAGCGTCAGATGTGTATAAGAGACAGNNNNNNNNGGTCAGTGGTCCCAAGTAGTCAC-3'	NGS
NGS-REV	5'-GTCTCGTGGGCTCGGAGATGTGTATAAGAGACAGACATGGGGTCCCCCACTATC-3'	NGS
Y_Adaptor-FW	5’-[PHO]CTCACCGCTCTTGTAGSNNNNNNNNCTGTCTCTTATACACATCTCCGAG*C-3’	GUIDE-Seq
Y_Adaptor-RV	5’-CTACAAGAGCGGTGAGT-3’	GUIDE-Seq
dsODN_FW	5’-[PHO]G*T*TTAATTGAGTTGTCATATGTTAATAACGGT*A*T-3’	GUIDE-Seq
dsODN_RV	5’-[PHO]A*T*ACCGTTATTAACATATGACAACTCAATTAA*A*C-3’	GUIDE-Seq
Enrich_Plus	5’-TCGTCGGCAGCGTCAGATGTGTATAAGAGACAGNNNGTTTAATTGAGTTGTCATATGTTAATAACGG-3’	GUIDE-Seq
Enrich_RV	5’-TCGTCGGCAGCGTCAGATGTGTATAAGAGACAGNNNCCGTTATTAACATATGACAACTCAATTAAAC-3’	GUIDE-Seq
2nd_R1	5’-AATGATACGGCGACCACCGAGATCTACACNNNNNNNNTCGCTCGTCGGCAGCGTC-3’	GUIDE-Seq
2nd_R2	5’-CAAGCAGAAGACGGCATACGAGATNNNNNNNNGTCTCGTGGGCTCGG-3’	GUIDE-Seq

‘NNNNNNNN’ indicates barcode sequence.
‘[PHO]’ represents 5’ phosphorylation.
‘*’ indicates a phosphorothioate linkage.

Supplementary Table 2. Mutations of hemophilia B from the data base of European Association for Haemophilia and Allied Disorders.

Mutation		Number	Ratio (%)
Point mutation		4,151	89.1
	A>C	71	1.5
	A>G*	294	6.3
	A>T	69	1.5
	C>A	111	2.4
	C>G	89	1.9
	C>T*	1,061	22.8
	G>A*	1,410	30.3
	G>C	172	3.7
	G>T	273	5.9
	T>A	121	2.6
	T>C*	363	7.8
	T>G	117	2.5
Deletion		406	8.7
Duplication		60	1.3
Indel		22	0.5
Insertion		14	0.3
Complex		4	0.1
Total		4,657	100

*The “C to T” or “A to G” base editor could repair the mutations.
