# Supplementary material for: PAM-flexible Cas9-mediated base editing of a hemophilia B mutation in induced pluripotent stem cells
Source: Commun Med (Lond). 2023 Apr 19;3:56. doi: 10.1038/s43856-023-00286-w (PMC10115777; doi:10.1038/s43856-023-00286-w)
Supplement: Supplementary file 2 — Description of Additional Supplementary Files [file 43856_2023_286_MOESM2_ESM.pdf]

Description of Additional Supplementary Files

File Name: Supplementary Data 1
Description: The results of GUIDE-Seq analysis.

File Name: Supplementary Data 2
Description: Data underlying the main figures and Supplemental information.
