# Supplementary material for: PAM-flexible Cas9-mediated base editing of a hemophilia B mutation in induced pluripotent stem cells
Source: Commun Med (Lond). 2023 Apr 19;3:56. doi: 10.1038/s43856-023-00286-w (PMC10115777; doi:10.1038/s43856-023-00286-w)
Supplement: Supplementary file 6 — Reporting Summary [file 43856_2023_286_MOESM6_ESM.pdf]

nature research

Corresponding author(s): Tsukasa Ohmori

Last updated by author(s): 4/3/2023

Reporting Summary

Nature Research wishes to improve the reproducibility of the work that we publish. This form provides structure for consistency and transparency in reporting. For further information on Nature Research policies, see our Editorial Policies and the Editorial Policy Checklist.

Statistics

For all statistical analyses, confirm that the following items are present in the figure legend, table legend, main text, or Methods section.

n/a	Confirmed	
☐	☒	The exact sample size (n) for each experimental group/condition, given as a discrete number and unit of measurement
☐	☒	A statement on whether measurements were taken from distinct samples or whether the same sample was measured repeatedly
☐	☒	The statistical test(s) used AND whether they are one- or two-sided<br>*Only common tests should be described solely by name; describe more complex techniques in the Methods section.*
☒	☐	A description of all covariates tested
☐	☒	A description of any assumptions or corrections, such as tests of normality and adjustment for multiple comparisons
☐	☒	A full description of the statistical parameters including central tendency (e.g. means) or other basic estimates (e.g. regression coefficient) AND variation (e.g. standard deviation) or associated estimates of uncertainty (e.g. confidence intervals)
☐	☒	For null hypothesis testing, the test statistic (e.g. F, t, r) with confidence intervals, effect sizes, degrees of freedom and P value noted<br>*Give P values as exact values whenever suitable.*
☒	☐	For Bayesian analysis, information on the choice of priors and Markov chain Monte Carlo settings
☒	☐	For hierarchical and complex designs, identification of the appropriate level for tests and full reporting of outcomes
☒	☐	Estimates of effect sizes (e.g. Cohen's d, Pearson's r), indicating how they were calculated

Our web collection on statistics for biologists contains articles on many of the points above.

Software and code

Policy information about availability of computer code

Data collection	We used the softwares as below;<br>Gel imaging: ImageQuant LAS 4000 (Cytiva)<br>Immunofluorescence Imaging: BIOREVO BZ-9000 analysis software (KEYENCE) and<br>LAS AF software (Leica Microsystems)<br>Flow cytometry analysis: Diva software version 8.0.1 (BD biosciences)<br>Quantitative PCR: QuantStudio 12K Flex Software (Life Technologies)
Data analysis	We used the softwares as below;<br>Statistic analysis: Graph Pad Prism 9 software (Graph Pad Software)<br>Sequence analysis: SnapGene Version 5.1.5 (SnapGene Software)<br>Flow cytometry data analysis: FlowJo Version 10.6.2<br>Figure generation: Adobe Illustrator 2021 (Adobe)<br>illustration: BioRender.com

For manuscripts utilizing custom algorithms or software that are central to the research but not yet described in published literature, software must be made available to editors and reviewers. We strongly encourage code deposition in a community repository (e.g. GitHub). See the Nature Research guidelines for submitting code & software for further information.

Data

Policy information about availability of data

All manuscripts must include a data availability statement. This statement should provide the following information, where applicable:

- Accession codes, unique identifiers, or web links for publicly available datasets
- A list of figures that have associated raw data
- A description of any restrictions on data availability

Source data underlying the Figures are provided as Supplementary Data 2 file. The original data obtained from the current study are available from the corresponding author on reasonable request.

Field-specific reporting

Please select the one below that is the best fit for your research. If you are not sure, read the appropriate sections before making your selection.

☒ Life sciences ☐ Behavioural & social sciences ☐ Ecological, evolutionary & environmental sciences

For a reference copy of the document with all sections, see nature.com/documents/nr-reporting-summary-flat.pdf

Life sciences study design

All studies must disclose on these points even when the disclosure is negative.

Sample size	Sample sizes were determined based on pilot studies and analysis of statistical signficance.
Data exclusions	We did not exclude data.
Replication	To ensure reproducibility of our findings, we collected the data from at least three independent experiments in vitro. For in vivo experiments, we did not replicate with the same design, but we used 4 animals for each condition and found the same results. Exact for each experiment reported in the figure legends.
Randomization	Randomization was not used in vitro and in vivo experiments.
Blinding	Investigators were not blinded during data collection or analysis because planing and analysis of the studies was performed by the same personnel.

Reporting for specific materials, systems and methods

We require information from authors about some types of materials, experimental systems and methods used in many studies. Here, indicate whether each material, system or method listed is relevant to your study. If you are not sure if a list item applies to your research, read the appropriate section before selecting a response.

Materials & experimental systems

n/a	Involved in the study	
☐	☒	Antibodies
☐	☒	Eukaryotic cell lines
☒	☐	Palaeontology and archaeology
☐	☒	Animals and other organisms
☒	☐	Human research participants
☒	☐	Clinical data
☒	☐	Dual use research of concern

Methods

n/a	Involved in the study	
☒	☐	ChIP-seq
☐	☒	Flow cytometry
☒	☐	MRI-based neuroimaging

Antibodies

Antibodies used

Antibody details were described in method section.
anti-NANOG mouse monoclonal antibody (clone 23D2-3C6; BioLegend)
anti-OCT4 mouse monoclonal antibody (clone 3A2A20; BioLegend)
anti-mouse IgG conjugated with AlexaFluor 594 (ThermoFIsher Scientific)
PE-conjugated anti-human SSEA-4 monoclonal antibody (clone MC-813-70; BioLegend)
Alexa Fluor 488-conjugated anti-human Tra-1-60-R monoclonal antibody (clone TRA-1-60-R; BioLegend)
anti-human FIX goat polyclonal antibody conjugated with biotin (GAFIX-AP, Affinity Biologicals)
anti-Giantin rabbit polyclonal antibody (Poly19087; BioLegend)
streptavidin-conjugated with AlexaFluor 594 (ThermoFisher Scientific)
anti-rabbit goat antibody conjugated with AlexaFluor 488 (Thermo Fisher Scientific)

	anti-human FIX sheep polyclonal antibody (CL20039AP; CEDARLANE)<br>anti-human FIX goat antibody conjugated with horseradish peroxidase (GAFIX-HRP, Affinity Biologicals)
Validation	All antibodies were commercially available and validated by the suppliers, with cited references if applicable and accurately represented expression patterns.

Eukaryotic cell lines

Policy information about cell lines

Cell line source(s)	We generated all iPSCs in this paper from a healthy donor and a patient with severe hemophilia B after obtaining written informed consent. HEK293 cells were obtained from ATCC.
Authentication	The morphologies, self-renewal and differentiation abilities were tested and described partially in Figure 2D and 2E.
Mycoplasma contamination	Our cells were not tested for mycoplasma contamination.
Commonly misidentified lines<br>(See ICLAC register)	No commonly misidentified cell lines were used in this study.

Animals and other organisms

Policy information about studies involving animals; ARRIVE guidelines recommended for reporting animal research

Laboratory animals	NOG (NOD.Cg-Prkdcscid Il2rgtm1Sug/Jic) and TK-NOG [NOD.Cg-Prkdcscid Il2rgtm1SugTg (Alb-UL23)7-2/ShiJic] mice were purchased from In-Vivo Science Inc. (Tokyo, Japan). Knock-in mice expressing F9 cDNA were generated as described in Material and Methods.
Wild animals	No wild animals were used in this study.
Field-collected samples	No field-collected samples were used in this study.
Ethics oversight	All animal experiments were performed in compliance with the Institutional Animal Care and Concern Committee at Jichi Medical University and animal care was carried out in accordance with the committee's guidelines.

Note that full information on the approval of the study protocol must also be provided in the manuscript.

Flow Cytometry

Plots

Confirm that:

☒ The axis labels state the marker and fluorochrome used (e.g. CD4-FITC).

☒ The axis scales are clearly visible. Include numbers along axes only for bottom left plot of group (a 'group' is an analysis of identical markers).

☒ All plots are contour plots with outliers or pseudocolor plots.

☒ A numerical value for number of cells or percentage (with statistics) is provided.

Methodology

Sample preparation	iPSCs were dissociated with TrypLE-Select (Thermofisher Scientific) and resuspended in PBS containing 0.5% bovine serum albumin and 2 mM EDTA. Then, the cells were incubated with antibodies on ice for 30 min. After staining, we washed the cells twice using PBS and analyzed after adding BD Cell Viability kit.
Instrument	LSR-Fortessa X-20 with 488 nm□640 nm□405 nm□561 nm lasers (BD Bioscience, Franklin Lakes, NJ)
Software	Diva software version 8.0.1 (BD biosciences) for collecting data and FlowJo Version 10.6.2 for data analysis.
Cell population abundance	We did not concentrate the cell population.
Gating strategy	First, we used FSC-H vs FSC-W to select singlets. We then collect the viable cells as 7AAD negative populations. We determined each positive populations compared with negative controls.

☐ Tick this box to confirm that a figure exemplifying the gating strategy is provided in the Supplementary Information.
